# An assessment of student satisfaction with peer teaching of clinical communication skills

**DOI:** 10.1186/1472-6920-14-217

**Published:** 2014-10-13

**Authors:** Jonathan KA Mills, William J Dalleywater, Victoria Tischler

**Affiliations:** Queens Medical Centre, University of Nottingham Medical School, Nottingham, UK; London College of Fashion, University of the Arts London, London, UK

**Keywords:** Communication skills, Peer-teaching, Medical education

## Abstract

**Background:**

Peer teaching is now used in medical education with its value increasingly being recognised. It is not yet established whether students differ in their satisfaction with teaching by peer-teachers compared to those taught by academic or clinical staff. This study aimed to establish satisfaction with communication skills teaching between these three teaching groups.

**Methods:**

Students participated in a role-play practical facilitated either by clinicians, peer-teachers or non-clinical staff. A questionnaire was administered to first-year medical students after participating in a communication skills role-play session asking students to evaluate their satisfaction with the session. Data were analysed in SPSS 20.

**Results:**

One hundred and ninety eight students out of 239 (83%) responded. Students were highly satisfied with the teaching session with no difference in satisfaction scores found between those sessions taught by peers, clinical and non-clinical staff members. 158 (80%) considered the session useful and 139 (69%) strongly agreed tutors facilitated their development. There was no significant difference in satisfaction scores based on tutor background.

**Conclusions:**

Satisfaction is as high when tutored by peer-teachers compared to clinicians or non-clinical staff. Constructive feedback is welcomed from a range of personnel. Final-year students could play an increasing role in the teaching of pre-clinical medical students.

## Background

Near-peer teaching (defined as a student or junior doctor one or more years senior to another student on the same course of medical training) is becoming increasingly popular [1, 2] and affords many benefits to faculty and students alike, from being cost-effective to developing the student as a teacher [3]. Medical students who teach their junior peers (those in the first or second year of the medical course) have reported positively on the experience, particularly in developing confidence, knowledge and clinical skills [4–6]. Peer teaching can be beneficial to introduce to students to topics that may not be covered well in the curriculum [7], such as anatomy [8], clinical skills examination preparation [9], procedural workplace based skills [10] and prescribing [11]. Peer-teaching can result in attainment results comparable with those taught by clinical lecturers and associate professors [10].

Peer teaching however can be viewed positively or negatively depending on assessment characteristics and teaching environment by students receiving it, particularly if there are elements of peer assessment involved [12, 13]. Peer teaching, when used with other teaching methods offers added value in fostering cooperation and social interaction amongst students [14]. Quality assurance within higher education in the United Kingdom is placing increasing emphasis on student satisfaction, particularly the National Student Survey [15] held each year asking students to rate their university and course, asking questions on multiple aspects of the student experience, not just academic teaching quality. Satisfaction may relate to a stable personal-environmental relationship, with perceived quality of services offered relating to satisfaction outcomes [16]. A systematic review by Yu et *al*. [17] found studies on peer-teaching focused on formative assessment outcomes in written and practical assessments. Their study found peer teaching achieved short-term learner outcomes comparable to faculty-based teaching with the student-teaching reinforcing their own learning [17]. In a problem-based curriculum, peer-teachers who facilitated small group tutorials were perceived as better in delivering feedback, empathising with student difficulties, creating a better tutorial atmosphere, and gaining support from learners compared to groups facilitated by a faculty member [18]. There is however limited evidence on how satisfied students are with peer teaching compared to teaching delivered by a faculty member, and how receptive students are to feedback from near-peer student teachers on their communication skills.

This study aims to add to the literature by comparing satisfaction amongst medical students taught and given feedback from a senior peer-teacher, with those taught and given feedback from non-clinical or clinical facilitators.

## Methods

### Communication skills practical- before the session

Role-play facilitators were given training in communication skills by the module convenor on how to deliver the session and facilitate the feedback process prior to the timetabled sessions. Facilitators were experienced tutors in clinical and professional development who regularly teach students across the years, with clinicians working in primary care. Non-clinical facilitators were academics working in the Division of Psychiatry and Applied Psychology. Near-Peer teachers had completed peer-teaching training within the ‘early clinical and professional development’ module and expressed an interest in teaching. Training included the communication components considered for an effective clinical consultation as stated in the UK Consensus for the content of clinical communication curricula [19]. The use of Agenda-Led Outcome Based Analysis (ALOBA) method was promoted which encourages self-reflection, involving the group, providing feedback, and proposing alternatives in a non-judgmental way [20].

All First year medical students (n= 239) had a timetabled lecture in advance of the practical informing them of the structure of the role play session, the process for providing peer-feedback, the ground rules for a constructive and safe learning environment and information on this questionnaire study. Informed consent was obtained from participants, with students having the opportunity to decline to participate. Ethical approval was not required, as this was part of routine and pre-existing course evaluation.

The students also received teaching on the theoretical aspects of effective communication skills, question styles and basic interview structure using the Calgary Cambridge guide used by many medical schools [21].

### Participants

The students were first year undergraduates on a five year medical course who undertook a mandatory small group teaching session on communication skills using DVD-recorded simulated consultations, which did not contribute to grades awarded for the module. All facilitators (Academic staff, clinicians and peer-teachers) were randomly allocated to seminar groups having received training and guidance on the format of the session, how to use the recording equipment and how to provide feedback to students. A total of 24 seminar groups, each of approximately 10 students participated in the teaching session during the first semester of their undergraduate medical degree, ran over 8 periods within 3 weeks.

### The communication skills practical

Students were required to practice their communication skills with another student using a simulated consultation involving ‘doctor’ and ‘patient’ roles from scenarios provided. The objectives sought to encourage self-reflection on communication skills (both verbal and non-verbal), develop skills of providing feedback to others and empathise with patients. Volunteer students played the ‘patient’ roles. These included simulated patients presenting with chest pain, symptoms of post natal depression and a sexually transmitted infection. All consultations were DVD-recorded and watched back by the entire group after which feedback was provided to those involved. Students watching the consultation provided verbal feedback on different aspects of communication including verbal and non-verbal elements, giving examples of good practice and areas which required improvement. Each student playing the ‘doctor’ role undertook self-appraisal first, followed by ‘patient’ feedback, peer-feedback and finally facilitator feedback. This process was repeated until all students had the opportunity to take the ‘doctor’; role and to receive feedback on their communication skills.

### Data collection

Students were asked at the end of the session to provide feedback about the session on a questionnaire developed by the authors, which had been piloted successfully the previous year to evaluate their satisfaction with the session. The questionnaire consisted of five closed questions, three using a Likert scale and two requiring dichotomous responses on the opportunity to role play and whether the session kept to time, in addition to the opportunity to provide free text comments (see Table 1). The questionnaire was distributed and collected by a course administrator who had no part in the tutorials. The questions aimed to assess whether student-learners felt the session ran smoothly, their ability to contribute to discussions and whether they felt they benefited from this role-play teaching session. It also aimed to determine whether the facilitator was evaluated differently according to their role. The free text comments box was provided to allow student-learners the opportunity to provide comments (positive and negative) if they chose to. The questions were chosen to determine if students felt able to participate, felt they developed professionally and if they found the format of the tutorial useful to facilitate their learning. The questions were considered and worded neutrally so students could indicate their level of agreement with them without bias following the pilot the previous year.

**Table 1:** **Teaching satisfaction questionnaire**

Date	Seminar group		Tutor	
1) The session kept to time			Yes	No
2) I had the opportunity to role play			Yes	No
3) I had the opportunity to contribute to the discussion				
Strongly Disagree		Neutral	Strongly Agree	
1	2	3	4	5
4) The tutor facilitated my communication skills development				
Strongly Disagree		Neutral	Strongly Agree	
1	2	3	4	5
5) I found the session useful				
Strongly Disagree		Neutral	Strongly Agree	
1	2	3	4	5
Any comments?				

### Data analysis

Data was analysed in SPSS 20 using Analysis of Variance to assess differences between facilitator groups and student responses. Free text was also collected to analyse comments made by students about the teaching. Two raters independently read through the comments and suggested recurring themes, e.g. comments such as “I felt I learnt a lot” and “very informative” were considered under the theme of finding the session “useful to learning”. The raters then independently read through the comments and assigned them to agreed themes.

## Results

A total of 198 out of a potential 239 responses were received across 20 seminar groups (83% response rate), 4 groups did not return questionnaires. Ninety seven (49%) were taught by non-clinical academic staff, 60 (30%) taught by clinicians and 41 (21%) responses were received from those taught by final year peer-teachers. Students were asked to rate how well they felt able to contribute to the discussion. 160 (81%) felt strongly able to contribute, with only 2 (1%) strongly feeling they could not contribute. Students were asked whether they felt the tutor facilitated their development, 137 (69%) strongly agreed that the tutor had facilitated their development, with only 3 (1.5%) strongly disagreeing Students were asked to rate the usefulness of the session, with 158 (80%) rating the session as very useful, whilst only 3 (1.5%) Strongly disagreeing that the session was useful. These results were then contrasted amongst the three groups of facilitators. Analysis of Variance with Bonferroni correction did not demonstrate significant differences in satisfaction measures between the three groups (see Table 2).

**Table 2:** **Analysis of variance between the three groups**

Dependent variable	(I) facilitator	(J) facilitator	Mean difference (I-J)	Std. error	Sig.		95% confidence interval
						Lower bound	Upper bound
I had the opportunity to contribute to the discussion	Clinician	Academic staff	.06804	.09854	1.00	-.1699	.3060
		Peer- teacher	.04390	.12157	1.00	-.2497	.3375
	Academic staff	Peer- teacher	-.02414	.11176	1.00	-.2940	.2457
I found the session useful	Clinician	Academic staff	.03866	.10361	1.00	-.2115	.2889
		Peer- teacher	-.05488	.12783	1.00	-.3636	.2538
	Academic staff	Peer- teacher	-.09354	.11752	1.00	-.3773	.1902
The tutor facilitated my communication skills development	Clinician	Academic staff	.21392	.10613	.136	-.0424	.4702
		Peer- teacher	.01829	.13093	1.00	-.2979	.3345
	Academic staff	Peer- teacher	-.19562	.12037	.317	-.4863	.0950

Positive comments received in the free-text feedback related to 6 themes, how useful the session was (n= 19, 10%), How valued feedback was (n= 12, 6%), how positive the experience was (n= 11, 6%), how anxiety was relieved (n= 9, 5%), request for more sessions (n= 5, 3%) and benefits to working in a group (n= 3, 2%):

*“I had been apprehensive beforehand about watching myself back on the video but in the event I was made to feel fairly comfortable about it” (Academic staff)*

*“A useful experience, less scary than expected” (Clinician)*

*“Well structured, good at engaging with us, gave constructive feedback to help improve communication skills” (Peer-teacher)*

*“Felt involved in the whole session, feel more confident about my communication skills and how to develop them, very good feedback, would recommend to other groups” (Peer-teacher)*

*“Very useful, I learnt a lot in a short space of time. The tutor was very supportive” (Academic staff)*

*“I was anxious at first but facilitator was very supportive, went through what was good and what needed improvement, and learnt clearly what is expected in a clinical history and communication skills required to get it. I feel a lot more confident about communication skills. Thank you” (Peer-teacher)*

*“Would like to do this again in a couple of months!” (Academic staff)*

Negative Comments related to technical faults (n= 3, 2%), request for more information in advance (n= 2, 1%) and inappropriate choice of venue (n= 1, 1%).

*“Unfortunate choice of room” (Academic staff)*

*“Maybe give a little more info about the structure of the interview in advance. Also lengthy technical fault at start” (Academic staff)*

*“Enjoyed the session, only ran over because of equipment failure” (Peer-teacher)*

## Discussion

This study aimed to examine satisfaction between those taught by final year peer-teachers and those taught more traditionally by clinical or academic staff. The results demonstrated high satisfaction amongst first years with the practical communication skills session, regardless of tutor and show that near-peer teaching is valued by students.

Role play is a common method used to introduce students to consultation and communication skills [22–24]. However, some students dislike such sessions because they feel unable to participate because they feel embarrassed [25]. The students who participated in our sessions enjoyed the experience, finding it a valuable learning experience. Perhaps the tactful approach of using role-play, where all students are expected to participate alleviates some of the findings of Stevenson and Sander [25], or students can appreciate the relevance of developing consultation and communication skills to their future practice. In a study by Nestel and Kidd [26], first year students who went on to interview simulated patients following a tutorial on interviewing, found that simulated patients were significantly more satisfied with interviews from the students facilitated by peer-teachers compared to those facilitated by clinical-teachers. Comments received in the free-text feedback were encouraging, that generally, students found the session useful. A number of students experienced some initial anxiety, perhaps relating to being filmed or performing in front of their peer-group, though reassuringly their anxieties subsided during the role-play session. We were pleased that a handful of students also requested an increase in the number of such tutorials. Our findings that students valued role-play as a means to facilitate their learning are consistent with Nestel and Tierney [27], who found around 80% of students valued role-play. The finding of no difference in satisfaction between who the facilitator was (peer-teacher, clinician or academic staff member) suggests students valued feedback from any source they felt was credible, and not just limited to academic staff. The literature suggests first hand feedback, given contemporaneously is important [28] in helping students develop.

In addition to the benefits to the junior students and peer-teachers of developing their communication and teaching skills, peer-teaching may also allow medical schools to deploy their resources more effectively. With resources limited within medical faculties, providing faculty-educators may prove to be a struggle for increasing student demand for labour intensive small group tutorials, not just in clinical communication skills but other medical education disciplines such as anatomy. In view of the manifold benefits peer teaching may bring, it is becoming a more utilised resource; it is cost effective and provides opportunities for senior students to develop their skills as a teacher [3]. Increasing use of peer-teachers may provide an option for medical schools in addressing increasing demands from students, and moreover be useful to the student-teachers themselves [29].

### Implications

With students in higher education facing increasing tuition fees, and demands on universities to provide better ‘value for money’ , satisfaction with teaching is likely to become increasingly important. It is becoming more commonplace to expose first and second year medical students to clinical encounters, such as through General Practice or hospital wards as part of their clinical professional development and allow them the opportunity to practice clinical skills taught from the beginning of medical school. However, such clinical experience would be limited without the opportunity to practice clinical consultation and communication skills.

With increasing demands for future clinicians of all grades to teach their junior peers, and provide education to their patients, peer-teaching is becoming an increasingly important aspect to be included in the medical curriculum [30]. Peer-teaching strengthens the student-teacher’s knowledge and skills, with learning being reinforced by teaching the material to others [31].

### Strengths and weaknesses

The strengths of this study were it sampled a large cohort of undergraduates, and compared three different groups; final year peer-teachers, clinicians and faculty staff. Previous studies have tended to focus on student-teachers and faculty-teachers, but including clinicians improves the strength of the study that feedback was valued irrespective of the role of the individual who provided it. This study was limited in that it only looked at first year medical students after their first experience in medical school with role-play. Sessions ran across a 3 week period, so students may have heard feedback from peers influencing their expectations, though satisfaction was consistent between groups so effects of discussion within the year was likely negligible. No data was collected on demographics, though the high response rate would suggest the results represent a typical demographic of undergraduate medical students at a UK university; typically aged 18–20, slightly more females, with a diverse ethnic group.

### Further research

Future studies may explore the relationship between experiences of peer, clinician or academic teaching has an impact on summative assessment of clinical communication skills.

## Conclusion

Our results add to the evidence on peer teaching as students are just as satisfied with peers teaching them as with clinicians or academic staff, Final-year medical students could therefore provide a cost-effective and valuable learning resource for less experienced peers.
